# Comparing the Efficiency of Kerman Province Towns in Acquiring Human Development Index via Data Envelopment Analysis

**Published:** 2012-04-01

**Authors:** A Saber Mahani, M Hadian, H Ghaderi, M Barouni, A Shakibaei, M A Bahrami

**Affiliations:** 1 Student in Health Economics, Tehran University of Medical Sciences, Tehran, Iran; 2 Department of Health Economics, Tehran University of Medical Sciences, Tehran, Iran; 3 Department of Health Economics, Kerman University of Medical Sciences, Kerman, Iran; 4 Department of Healthcare Management, Shahid Sadoughi University of Medical Sciences, Yazd, Iran

**Keywords:** Human development index, Data envelopment analysis, Efficiency

Dear Editor,

The human development index (HDI) is a composite index calculated on the basis of three socioeconomic indicators that reflect three major dimensions of human development: longevity, educational attainment and standard of living to sufficiently capture the multidimensionality of human development. Longevity is measured by life expectancy at birth (LEB); educational attainment is measured by a weighted average of the adult literacy rate (ALR) and the combined gross educational enrolment ratios (GER). An adjusted gross domestic product (GDP) per capita, converted into US dollars on the basis of the purchasing power parity exchange rate (PPP USD), is used as a measure of a decent standard of living. For the components of the HDI, except of the GDP per capita, individual indices are calculated according to the general linear transformation:

*Index* = *actual value* - *fixed* min *imumvalue* / *fixed* max *imumvalue* - *fixed* min *mumvalue*

To construct the income index, the following nonlinear transformation is applied on GDP per capita, taking into account diminishing returns of higher incomes (utility adjustment):

*Income Index* = log *(actual GDP for capita)* - log *(min imum GDP for Capita)* / log *( fixed max imum value)* - *( fixed min imum value)*

The fixed minimum and maximum values of indicators are 25 and 85 years for LEB, 0% and 100% for ALR and GER and 100 and 40000 US$ for GDP.[1][2][3][4][5] Data envelopment analysis (DEA) is the leading technique for measuring the relative efficiency of decision making units on the basis of multiple inputs and outputs. The efficiency of a unit is defined as the weighted sum of its outputs divided by a weighted sum of its inputs and it is measured on a bounded ratio scale.

The weights for inputs and outputs are estimated by a linear program in the best advantage for each unit so as to maximize its relative efficiency. Basically, DEA provides a categorical classification of the units into efficient and inefficient ones by assuming either constant returns to scale (introduced by Charnes, Cooper and Rhodes named CCR model) or variable returns to scale (introduced by Banker, Charnes and Cooper named BCC model) for the inputs and outputs.[6][7][8][9] In this paper, we have been considered the assessment of Kerman Province towns technical efficiency in accessing HDI via DEA.

For this objective, in the descriptive study, we applied CCR and BCC models for assessing Kerman town’s technical efficiency in accessing HDI by using DEP2 software. Each town’s HDI was considered as output and the number of physicians for 1000 people (a proxy for life expectancy), educational staff rates (a proxy for educational attainment) and the employment rate of over 10 years working workers (a proxy for GDP) was considered as inputs for calculating the efficiency. All findings of study have been summarized in Table 1.

**Table 1:** HDI and Efficiency scores with BCC and CCR output oriented models in the years 2000 and 2007

**Township **	**HDI score **		**Efficiency score **				**Income ** **index **		**Health ** **index **		**Education ** **index **	
	**2000**	**2007**	**BCC** **2000**	**CCR** **2000**	**BCC** **2007**	**CCR** **2007**						
							**2000**	**2007**	**2000**	**2007**	**2000**	**2007**
Baft	0 . 549	0 . 649	0.942	0.810	0.980	0.968	0.38	0.52	0.69	0.73	0.57	0.69
Bardsir	0 . 543	0 . 653	1.000	1.000	1.000	1.000	0.36	0.49	0.73	0.76	0.52	0.69
Bam	0 . 550	0 . 651	0.850	0.750	0.906	0.777	0.36	0.47	0.71	0.75	0.57	0.73
Jiroft	0 . 496	0 . 653	0.764	0.713	1.000	1.000	0.30	0.52	0.66	0.70	0.52	0.73
Ravar	0 . 442	0 . 650	1.000	1.000	1.000	1.000	0.17	0.56	0.70	0.73	0.44	0.64
Rafsenjan	0 . 583	0 . 727	0.864	0.605	1.000	1.000	0. 40	0.68	0.76	0.79	0.58	0.69
Zarand	0 . 562	0 . 658	0.958	0.832	0.980	0.898	0.36	0.61	0.71	0.75	0.60	0.60
Sirjan	0 . 600	0 . 654	1.000	0.940	1.000	1.000	0.50	0.52	0.69	0.73	0.59	0.70
Share Babak	0 . 559	0 . 698	1.000	1.000	1.000	0.837	0.34	0.56	0.74	0.78	0.58	0.74
Kerman	0 . 558	0 . 665	1.000	1.000	0.930	0.914	0.34	0.51	0.67	0.70	0.65	0.77
Kahnouj	0 . 486	0 . 660	1.000	1.000	1.000	1.000	0.26	0.60	0.71	0.75	0.47	0.61
Mean of north township	0.549	0.669	0.970	0.898	0.986	0.952	-	-	-	-	** - **	**- **
Mean of south township	0 . 510	0.656	0.871	0.821	0.969	0.925	-	-	-	-	** - **	**- **

Based on the findings of study we concluded HDI score of all towns of Kerman Province have been improved in the year 2007 relative to year 2000. All towns were in the average range of HDI (0/5 to 0.8).[2] We can conclude that middle range scores of some towns including Bardsir, Ravar and Kahnouj resulted from input shortage but other towns scores showed some inefficiencies that can improve by more efficient use of inputs.
